# Effects of Sex on Early Outcome following Repair of Acute Type A Aortic Dissection: Results from The Nordic Consortium for Acute Type A Aortic Dissection (NORCAAD)

**DOI:** 10.1055/s-0039-1687900

**Published:** 2019-07-22

**Authors:** Raphaelle A. Chemtob, Vibeke Hjortdal, Anders Ahlsson, Jarmo Gunn, Ari Mennander, Igor Zindovic, Christian Olsson, Aldina Pivodic, Emma C. Hansson, Anders Jeppsson, Arnar Geirsson, Tomas Gudbjartsson

**Affiliations:** 1 Department of Thoracic and Cardiovascular Surgery, Heart and Vascular Institute, Cleveland Clinic, Cleveland, Ohio; 2 Department of Cardiothoracic and Vascular Surgery, Aarhus University Hospital, Skejby, Denmark; 3 Heart and vascular theme, Karolinska University Hospital, Stockholm, Sweden; 4 Department of Surgery, Heart Center, Turku University Hospital, University of Turku, Turku, Finland; 5 Department of Cardiothoracic Surgery, Heart Center, Tampere University Hospital, Tampere, Finland; 6 Department of Clinical Sciences, Skane University Hospital, Lund University, Lund, Sweden; 7 Department of Cardiothoracic Surgery, Skane University Hospital, Lund University, Lund, Sweden; 8 Statistiska Konsultgruppen, Gothenburg, Sweden; 9 Department of Cardiothoracic Surgery, Sahlgrenska University Hospital, Gothenburg, Sweden; 10 Department of Molecular and Clinical Medicine, Institute of Medicine, Sahlgrenska Academy, University of Gothenburg, Gothenburg, Sweden; 11 Section of Cardiac Surgery, Yale University School of Medicine, New Haven, Connecticut; 12 Department of Cardiothoracic Surgery, Landspitali University Hospital, Hringbraut, Reykjavik, Iceland

**Keywords:** aortic dissection, aortic operation, outcomes, surgery, complications, sex

## Abstract

**Background** Female sex is known to have increased perioperative mortality in cardiac surgery. Studies reporting effects of sex on outcome following surgical repair for acute Type A aortic dissection (ATAAD) have been limited by small cohorts of heterogeneous patient populations and have shown diverging results. This study aimed to compare perioperative characteristics, operative management, and postoperative outcome between sexes in a large and well-defined cohort of patients operated for ATAAD.

**Methods** The Nordic Consortium for Acute Type A Aortic Dissection study included patients with surgical repair of ATAAD at eight Nordic centers between January 2005 and December 2014. Independent predictors of 30-day mortality were identified using multivariable logistic regression.

**Results** Females represented 373 (32%) out of 1,154 patients and were significantly older (65 ± 11 vs. 60 ± 12 years, *p* < 0.001), had lower body mass index (25.8 ± 5.4 vs. 27.2 ± 4.3 kg/m ^2^ , *p* < 0.001), and had more often a history of hypertension (59% vs. 48%, *p* = 0.001) and chronic obstructive pulmonary disease (8% vs. 4%, *p* = 0.033) compared with males. More females presented with DeBakey class II as compared with males with dissection of the ascending aorta alone (33.4% vs. 23.1%, *p* = 0.003). Hypothermic cardiac arrest time (28 ± 16 vs. 31 ± 19 minutes, *p* = 0.026) and operation time (345 ± 133 vs. 374 ± 135 minutes, *p* < 0.001) were shorter among females. There was no difference between the sexes in unadjusted intraoperative death (9.1% vs. 6.7%, *p* = 0.17) or 30-day mortality (17.7% vs. 17.4%, *p* = 0.99). In a multivariable analysis including perioperative factors influencing mortality, no difference was found between females and males in 30-day mortality (odds ratio: 0.92, 95% confidence interval: 0.62–1.38, *p* = 0.69).

**Conclusions** This study found no association between sex and early mortality following surgery for ATAAD, despite females being older and having more comorbidities, yet also presenting with a less widespread dissection than males.

## Introduction

Acute Type A aortic dissection (ATAAD) is a lethal cardiovascular emergency for which surgical repair is essential. 1 Despite significant improvements in perioperative management over the past decades, surgery for ATAAD is still associated with high mortality and morbidity. 2 Several factors, such as advanced age and comorbidities including diabetes and chronic obstructive pulmonary disease (COPD), preoperative cardiogenic shock with or without cardiac tamponade, and longer cardiopulmonary bypass (CPB) time, have been suggested as independent predictors of poor outcome. 3 4 5

Sex differences have previously been shown to influence outcomes following cardiac surgery. 6 7 In the risk models of both the Society of Thoracic Surgeons score and the European System for Cardiac Operative Risk Evaluation, female sex is a risk factor for early mortality after cardiac surgery. 6 7 8 In spite of sex differences being well documented in patients undergoing cardiac surgery, there have been few reports that specifically compare outcomes for males and females following surgical repair for ATAAD. Of the reported studies, some have found female sex to be an independent predictor of mortality after surgical repair for ATAAD, 3 9 10 while others found no significant association between sex and outcome. 4 5 11 The previous studies have been limited by small cohorts of heterogeneous study samples and have shown diverging results. This report from The Nordic Consortium for Acute Type A Dissection (NORCAAD) constitutes one of the largest studies investigating the impact of sex differences on outcome following surgical repair for ATAAD. The aim of this study was to determine the impact of sex on preoperative characteristics, operative management, and postoperative outcomes in a large multicenter cohort of patients who underwent surgical treatment for ATAAD.

## Materials and Methods

The study is a part of NORCAAD, which includes eight centers in Denmark, Finland, Iceland, and Sweden. The study setup is further described elsewhere. 12 In brief, data were retrospectively collected for adult patients (> 17 years of age) who underwent surgery for ATAAD between January 1, 2005 and December 31, 2014 at the following centers: Aarhus University Hospital, Skejby, Denmark; Karolinska University Hospital, Stockholm, Sweden; Landspitali University Hospital, Reykjavik, Iceland; Orebro University Hospital, Orebro, Sweden; Sahlgrenska University Hospital, Gothenburg, Sweden; Skane University Hospital, Lund, Sweden; Tampere University Hospital, Tampere, Finland; and Turku University Hospital, Turku, Finland. A total of 194 clinical variables were retrospectively collected, including demographics, past medical history, preoperative medications, clinical symptoms at presentation, methods of diagnosis, operative variables, complications, bleeding and blood transfusion, laboratory values, and outcome data. The length of stay was recorded as days in the intensive care unit and in the general ward.

The study was approved by each of the participating institutions. Patients were deidentified prior to inclusion into the database. As individual patients were not identified, obtaining individual consent for the study was waived.

### Statistics

Data were examined for normal distribution using the Shapiro–Wilk test. Comparisons of two groups were performed with Mann–Whitney U test for continuous variables. Nonordered categorical variables were compared with Pearson's chi-squared test and dichotomous variables with Fisher's exact test. All tests were two-tailed and conducted at a 0.05 significance level. Data are presented as mean ± standard deviation, median with 25th to 75th percentile (interquartile range) where applicable or number (percentage). Independent predictors of mortality were identified with odds ratio (OR) with 95% confidence interval (CI) estimated with a multivariable logistic regression. All variables were adjusted for age. The variables included in the multivariate analysis were selected based on degree of significance ( *p* < 0.20) from the univariate and age-adjusted analysis. All statistical analyses were performed by a professional statistician using SAS Software version 9.4 (SAS Institute Inc., Cary, NC).

## Results

A total of 1,154 patients were included in the study, of which 373 (32%) were females and 781 (68%) were males. The mean age for the cohort was 61 ± 12 years. Poisson regression and Cox proportional hazard analysis are found in the Supplementary Tables 1 and 2 , respectively.

### Patient Characteristics

Patient characteristics are outlined in Table 1 . Females were significantly older (65 ± 11 vs. 60 ± 12 years, *p* < 0.001), had lower body mass index (BMI) (26 ± 5 vs. 27 ± 4 kg/m ^2^ , *p* < 0.001), and more often had a history of hypertension (58.9% vs. 48.3%, *p* = 0.001) and COPD (8.3% vs. 4.9%, *p* = 0.03) as compared with males. Furthermore, there was a trend toward increased rates of hypercholesterolemia (14.2% vs. 10.6%, *p* = 0.09) and preoperative pericardial tamponade (19.2% vs. 15.7%, *p* = 0.08) among females. Although within the normal range, females had lower preoperative creatinine levels than males (80 ± 28 vs. 106 ± 58 μmol/L, *p* < 0.001).

**Table 1:** Patient characteristics

Variables	Male ( *n* = 781)	Female ( *n* = 373)	*p* -Value
Age	59.9 (12.2) 62.0 (14.0; 86.0) *n * = 781	65.0 (11.3) 66.0 (18.0; 85.0) *n* = 373	< 0.0001
Age > 70 y	157 (20.1%)	134 (35.9%)	< 0.0001
Body mass index	27.2 (4.3) 26.2 (18.0; 47.7) *n* = 648	25.8 (5.4) 24.8 (11.4; 53.9) *n* = 308	< 0.0001
Smoking	242 (44.2%)	129 (43.0%)	0.80
Hypertension	377 (48.3%)	219 (58.9%)	0.0010
Hypercholesterolemia	82 (10.6%)	53 (14.2%)	0.092
Diabetes mellitus II	15 (1.9%)	11 (3.0%)	0.37
COPD	38 (4.9%)	31 (8.3%)	0.033
HxStroke	30 (3.9%)	17 (4.6%)	0.67
HxTIA	14 (1.8%)	3 (0.8%)	0.29
HxCKD	17 (2.2%)	4 (1.1%)	0.28
HxAfib	23 (6.8%)	4 (2.4%)	0.052
Marfan syndrome	33 (4.2%)	16 (4.3%)	1.00
Preoperative creatinine (µmol/L)	105.7 (57.9)95.0 (6.2; 1000.0) *n* = 635	80.5 (28.2)75.0 (36.0; 285.0) *n* = 324	< 0.0001
Euroscore II	10.5 (11.2)7.3 (1.0; 65.4) *n* = 219	9.35 (6.43)8.50 (0.62; 27.90) *n* = 84	0.35
LVEF TEE (%)	49.1 (8.3)50.0 (0.5; 74.0) *n* = 272	48.4 (8.6)50.0 (0.0; 70.0) *n* = 130	0.37
Cardiac arrest	39 (5.0%)	18 (4.8%)	1.00
Cardiogenic shock	161 (22.3%)	91 (26.1%)	0.19
Pericardial tamponade	120 (15.7%)	70 (19.2%)	0.17
*DeBakey class:*			
I	596 (76.9%)	247 (66.6%)	
II	179 (23.1%)	124 (33.4%)	0.0003
*Penn Class:*			
Penn Class A	462 (59.7%)	243 (65.7%)	
Penn Class B	182 (23.5%)	63 (17.0%)	
Penn Class C	85 (11.0%)	41 (11.1%)	
Penn Class B and C	45 (5.8%)	23 (6.2%)	0.092

Abbreviations: Afib, atrial fibrillation; CKD, chronic kidney disease; COPD, chronic obstructive pulmonary disease; Hx, history of; LVEF, left ventricular ejection fraction; SD, standard deviation; TEE, transthoracic echocardiogram; TIA, transient ischemic attack.

Note: Data are presented as *n* (%) or mean (SD) / median (min; max).

### Perioperative Characteristics

The perioperative characteristics are outlined in Tables 2 and 3 . The median time from symptom presentation to definitive surgical treatment was 2.3 (1.0; 7.0) hours and 2.9 (1.0; 6.7) hours in females ( *n* = 208) and males ( *n* = 441), respectively ( *p* = 0.72). However, data on time delay were missing in 165 (44%) females and 441 (56%) of males. Furthermore, females had shorter operation time (345 ± 133 vs. 374 ± 134.8 minutes, *p* < 0.001), CPB time (198 ± 77 vs. 212 ± 79 minutes, *p* < 0.001), aortic cross-clamp time (99 ± 54 vs. 109 ± 57 minutes, *p* < 0.001), and hypothermic circulatory arrest (HCA) time (28 ± 16 vs. 31 ± 19 minutes, *p* = 0.02) as compared with males. In addition, significantly fewer females compared with males had surgical repair with a biological aortic valve replacement (3.8% vs. 7.1%, *p* = 0.03) and less often underwent repair using a mechanical composite graft (10.2% vs. 21%, *p* < 0.001). Surgical repair with valve resuspension, however, was more common among females as compared with males (44.2% vs. 37.3%, *p* = 0.001).

**Table 2:** Intraoperative characteristics

Variables	Male ( *n* = 781)	Female ( *n* = 373)	*p* -Value
Operation time	373.6 (134.8) 344.0 (36.0; 1059.0) *n* = 627	344.8 (133.3) 310.0 (15.0; 1100.0) *n* = 295	0.0001
Cardiopulmonary bypass time	211.6 (79.0) 197.0 (0.0; 715.0) *n* = 714	197.7 (77.4) 178.5 (22.0; 500.0) *n* = 344	< 0.0001
Cardiac arrest time	30.9 (18.8) 27.0 (0.0; 185.0) *n* = 602	28.1 (16.5) 26.0 (0.0; 195.0) *n* = 300	0.026
Temperature at cardiac arrest	21.0 (4.9) 19.0 (11.0; 36.4) *n* = 694	20.8 (4.6) 19.5 (13.0; 35.8) *n* = 340	0.76
Cross-clamp time	109.2 (57.6) 96.0 (0.0; 363.0) *n * = 697	98.8 (53.9) 86.0 (1.0; 364.0) *n* = 331	0.0012
Biologic aortic valve	55 (7.1%)	14 (3.8%)	0.033
*Proximal repair:*			
AV resuspension + ascending graft	291 (37.3%)	164 (44.2%)	
AVR + ascending graft	25 (3.2%)	9 (2.4%)	
Ascending graft only	220 (28.2%)	124 (33.4%)	
Attempted	7 (0.9%)	4 (1.1%)	
Biological composite	43 (5.5%)	22 (5.9%)	
Mechanical composite	164 (21.0%)	38 (10.2%)	
Valve sparing root	24 (3.1%)	8 (2.2%)	
Ascending graft only prior AVR	6 (0.8%)	2 (0.5%)	0.0013
*Distal repair:*			
Ascending aorta	543 (70.3%)	269 (73.1%)	
Attempted	5 (0.6%)	5 (1.4%)	
Hemiarch	171 (22.2%)	77 (20.9%)	
Total arch	51 (6.6%)	15 (4.1%)	
Other	2 (0.3%)	2 (0.5%)	0.27
Perioperative MI	45 (5.9%)	26 (7.1%)	0.52
Intraoperative death	52 (6.7%)	34 (9.1%)	0.17

Abbreviations: AV, aortic valve; AVR, aortic valve replacement; MI, myocardial infarction; SD, standard deviation.

Note: Data are presented as *n* (%) or mean (SD) / median (min; max).

**Table 3:** Postoperative characteristics

Variables	Male ( *n* = 729)	Female ( *n* = 339)	*p* -Value
Reoperation bleeding	174 (23.9%)	56 (16.5%)	0.0050
Length of stay in ICU	6.11 (8.45)4.00 (0.00; 134.00) *n* = 568	5.23 (5.81)3.00 (0.00; 40.00) *n* = 275	0.17
Length of stay	12.5 (12.6)9.0 (0.0; 136.0) *n* = 492	12.9 (13.0)9.0 (0.0; 96.0) *n* = 211	0.88
Postoperative AF	289 (39.6%)	143 (42.2%)	0.66
Postoperative stroke	117 (16.0%)	60 (17.7%)	0.67
Postoperative TIA	35 (4.8%)	17 (5.0%)	1.00
Postoperative cardiac arrest	54 (7.4%)	24 (7.08%)	0.89
Death at discharge	128 (17.5%)	61 (17.9%)	1.00
30-day mortality	138 (18.9%)	65 (19.2%)	0.99

Abbreviations: AF, atrial fibrillation; ICU, intensive care unit; SD, standard deviation; TIA, transient ischemic attack.

Note: Data are presented as *n* (%) or mean (SD) / median (min; max).

### Outcome Measures

Surgical outcome and postoperative complications are shown in Table 3 . Reoperation for bleeding was less common among females than in males (16.5% vs. 23.9%, *p* = 0.005). No difference was observed in intraoperative death among females as compared with males (9.1% vs. 6.7%, *p* = 0.17), death at discharge (16.4% vs. 16.4%, *p* = 1.00), or 30-day mortality (17.4% vs. 17.7%, *p* = 0.99).

In a multivariable logistic regression analysis for 30-day mortality ( Table 4 ), no difference was found between females and males in 30-day mortality (OR: 0.92, 95% CI: 0.62–1.38, *p* = 0.69).

**Table 4:** Univariable and multivariable logistic regression analyses for 30-day mortality

				Univariable a			Adjusted b		Multivariable c	
Prediction variables	*n* missing	Value	*n* (%) of event	OR (95% CI)30-d mortality	*p* -Value	Area under ROC-curve (95% CI)	OR (95% CI)30-d mortality	*p* -Value	OR (95% CI)30-d mortality	*p* -Value
Gender	0	Male	138 (17.7%)							
		Female	65 (17.4)	0.98 (0.71–1.36)	0.92	0.50 (0.47–0.54)	0.90 (0.65–1.25)	0.53	0.92 (0.62–1.38)	0.69
Age (OR per 5 units)	0	14.0–< 58.0	54 (14.4)							
		58.0–< 68.0	65 (17.0)							
		68.0–86.0	84 (21.2)	1.09 (1.02–1.16)	0.011	0.57 (0.52–0.61)	1.09 (1.02–1.16)	0.011	1.06 (0.98–1.15)	0.15
Age > 70 y	0	No	141 (16.3)							
		Yes	62 (21.3)	1.39 (0.99–1.94)	0.055	0.53 (0.50–0.57)	1.03 (0.65–1.64)	0.88		
BMI (OR per 5 units)	198	11.39–< 24.49	39 (12.3)							
		24.49–< 27.80	49 (15.4)							
		27.80–53.88	52 (16.2)	1.18 (0.99–1.41)	0.069	0.55 (0.50–0.60)	1.22 (1.02–1.47)	0.031		
Smoking	306	No	70 (14.7)							
		Yes	55 (14.8)	1.01 (0.69–1.48)	0.95	0.50 (0.45–0.55)	1.03 (0.70–1.51)	0.89		
Hypertension	2	No	82 (14.7)							
		Yes	120 (20.1)	1.46 (1.07–1.98)	0.017	0.55 (0.51–0.58)	1.39 (1.02–1.89)	0.040	1.22 (0.83–1.78)	0.31
Hypercholesterolemia	8	No	171 (16.9)							
		Yes	28 (20.7)	1.29 (0.82–2.01)	0.27	0.51 (0.49–0.54)	1.20 (0.76–1.88)	0.44		
Diabetes	5	No	191 (17.0)							
		Yes	11 (42.3)	3.58 (1.62–7.91)	0.0016	0.52 (0.50–0.54)	3.34 (1.51–7.41)	0.0030	3.57 (1.15–11.05)	0.027
COPD	4	No	181 (16.7)							
		Yes	21 (30.4)	2.18 (1.27–3.72)	0.0046	0.53 (0.50–0.55)	2.02 (1.18–3.48)	0.011	1.99 (1.02–3.85)	0.043
HxStroke	3	No	191 (17.3)							
		Yes	11 (23.4)	1.46 (0.73–2.92)	0.28	0.51 (0.49–0.53)	1.36 (0.68–2.74)	0.38		
HxTIA	4	No	199 (17.6)							
		Yes	3 (17.6)	1.01 (0.29–3.53)	0.99	0.50 (0.49–0.51)	0.94 (0.27–3.32)	0.93		
HxCKD	4	No	197 (17.4)							
		Yes	5 (23.8)	1.48 (0.54–4.08)	0.45	0.50 (0.49–0.52)	1.59 (0.57–4.42)	0.37		
HxAfib	649	No	68 (14.2)							
		Yes	7 (25.9)	2.11 (0.86–5.18)	0.10	0.52 (0.49–0.56)	1.98 (0.80–4.93)	0.14		
Marfan syndrome	4	No	197 (17.9)							
		Yes	5 (10.2)	0.52 (0.20–1.33)	0.17	0.51 (0.50–0.52)	0.65 (0.25–1.68)	0.37		
Preoperative creatinine (µmol/L) (OR per 10 units)	195	6.2–< 79.0	37 (11.5)							
		79.0–< 102.0	41 (12.9)							
		102.0–1000.0	76 (24.0)	1.07 (1.03–1.11)	0.0007	0.61 (0.56–0.66)	1.07 (1.03–1.11)	0.0007		
Euroscore II (OR per 5 units)	851	0.62–< 4.10	8 (7.9)							
		4.10–< 11.10	19 (18.4)							
		11.10–65.44	30 (30.3)	1.26 (1.11–1.43)	0.0003	0.67 (0.60–0.75)	1.22 (1.07–1.40)	0.0025		
LVEF TEE (%) (OR per 5 units)	752	0.0–< 50.0	26 (19.4)							
		50.0–< 55.0	12 (8.9)							
		55.0–74.0	13 (9.8)	0.78 (0.67–0.91)	0.0011	0.62 (0.52–0.71)	0.78 (0.67–0.91)	0.0011		
Cardiac arrest	4	No	180 (16.5)							
		Yes	22 (38.6)	3.19 (1.83–5.56)	< 0.0001	0.54 (0.51–0.56)	3.18 (1.82–5.55)	< 0.0001	1.64 (0.82–3.30)	0.16
Hypotensive shock at presentation	84	No	126 (15.4)							
		Yes	65 (25.8)	1.91 (1.36–2.68)	0.0002	0.56 (0.53–0.60)	1.85 (1.32–2.61)	0.0004	0.76 (0.45–1.28)	0.30
Pericardial tamponade	26	No	145 (15.5)							
		Yes	55 (28.9)	2.23 (1.55–3.19)	< 0.0001	0.56 (0.53–0.60)	2.15 (1.50–3.09)	< 0.0001	1.17 (0.73–1.86)	0.52
DeBakey class	8	I	153 (18.1)							
		II	44 (14.5)	0.77 (0.53–1.10)	0.15	0.52 (0.49–0.56)	0.71 (0.49–1.03)	0.074		
Penn class	10	Penn class A vs. Penn class A	77 (10.9)	1.00			1.00		1.00	
		Penn class B vs. Penn class A	52 (21.2)	2.20 (1.49–3.24)	< 0.0001		2.24 (1.52–3.31)	< 0.0001	2.31 (1.47–3.65)	0.0003
		Penn class C vs. Penn class A	43 (34.1)	4.23 (2.73–6.55)	< 0.0001		4.18 (2.69–6.49)	< 0.0001	4.35 (2.38–7.97)	< 0.0001
		Penn class B and C vs. Penn class A	29 (42.6)	6.06 (3.55–10.36)	< 0.0001	0.66 (0.62–0.70)	6.37 (3.71–10.94)	< 0.0001	6.61 (3.24–13.52)	< 0.0001
CPB time (OR per 30 units)	96	0–< 168	43 (12.1)							
		168–< 219	43 (12.4)							
		219–715	90 (25.2)	1.23 (1.16–1.30)	< 0.0001	0.64 (0.59–0.69)	1.23 (1.16–1.30)	< 0.0001	1.22 (1.15–1.30)	< 0.0001
Cross-clamp time (OR per 10 units)	126	0–< 76	45 (13.0)							
		76–< 116	42 (12.4)							
		116–364	83 (24.3)	1.07 (1.04–1.10)	< 0.0001	0.59 (0.54–0.65)	1.07 (1.05–1.10)	< 0.0001		

Abbreviations: Afib, atrial fibrillation; BMI, body mass index; CI, confidence interval; CKD, chronic kidney disease; COPD, chronic obstructive pulmonary disease; CPB, cardiopulmonary bypass; Hx, history of; LVEF, left ventricular ejection fraction; OR, odds ratio; ROC, receiver operating characteristic; TEE, transthoracic echocardiogram; TIA, transient ischemic attack.

Note: *p* -Values, OR, and area under ROC-curve are based on original values and not on stratified groups. OR is the ratio for the odds for an increase of the predictor of one unit. Area under ROC-curve with 95% CI for multivariable model is equal to 0.75 (0.71–0.79).

a All tests were performed with univariable logistic regression.

b Adjusting for age using logistic regression.

c Multivariable logistic regression model including sex, age, hypertension, diabetes, COPD, cardiac arrest, hypotensive shock at presentation, pericardial tamponade, Penn class, and CPB time.

## Discussion

In this study, we found no sex-related difference in early survival among patients who had undergone surgical repair for ATAAD. Females represented one-third of the cohort and were on average 5 years older, had lower BMI, and had more frequently a history of hypertension and COPD. Furthermore, CPB and HCA times were shorter among females, who less frequently underwent aortic valve replacement, compared with males.

Female sex has previously been suggested as a risk factor in patients undergoing surgical repair for ATAAD. However, most previous reports have included small cohorts, often involving heterogeneous patient populations, and have shown diverging results. 3 9 10 A limited number of multicenter studies, including the International Registry of Acute Aortic Dissection (IRAD), have examined the association between sex and outcome following surgical repair for aortic dissection. In the IRAD registry, Nienaber et al 9 found a significantly higher in-hospital mortality among females as compared with males (30.1% vs. 21.0%). However, the study included a heterogeneous group of patients with Stanford Type A and B dissections that were treated either medically and/or surgically. When adjusting for Type A aortic dissection, females still presented worse outcome. The increased mortality among females was suggested to be due to worse preoperative characteristics and an increased time-delay from symptom presentation to diagnosis as compared with males, although no risk-adjusted analysis was presented.

In this study, we found a shorter delay in the time duration from symptom presentation until definitive treatment with surgery among females than that for males, yet not statistically significant. A shorter delay in diagnosis could indicate that females were more inclined to seek medical attention on recognition of symptoms. However, as for ischemic heart disease, females often present with more atypical symptoms of ATAAD, resulting in misdiagnosis and treatment for other more common conditions, such as acute coronary syndrome. 13 14 15 It is well defined that the mortality rate increases significantly with time in patients with ATAAD, and a delay in diagnosis can have fatal consequences. 14

In a recent study by Melvinsdottir et al 16 that included both surgical cases and those diagnosed at autopsy, 35% of all patients with ATAAD died prior to hospital arrival. In consistency, another study reported that 49% of patients with ATAAD had died before hospital assessment, whereof 61% were females. 17 Death prior to hospital arrival may have influenced our results as we did not include these patients in this study, thus possibly underestimating the incidence of ATAAD and early fatality. Furthermore, females are found to be older than males at the onset of ATAAD and therefore may have been more prone to medical management as opposed to surgery. 18

The study by Lee et al investigated operative management and outcomes of 2,983 patients with ATAAD in North America. They found that both age above 70 years and female sex were independent predictors of mortality, in spite of the higher occurrence of ATAAD among males in their study. Nevertheless, the mean age for females and males was not reported, and the primary aim was not to study the effects of sex on outcome. 3 In most previous studies, the occurrence of ATAAD has consistently been lower among females (with a ratio of 1:2 or 1:3), who have also been found to be older with an average age difference of 7 to 11 years 4 5 9 10 11 16 and have lower BMI as compared with males. 4 5 In consistency with previous studies, females were also significantly older, had lower BMI, and more frequently had a history of hypertension and COPD in the present cohort. Females also presented with significantly lower preoperative creatinine, although within the normal range. Interestingly, and despite these variances in patient characteristics, we found no difference in mortality among sexes. When adjusting for perioperative factors in a multivariable analysis, no difference was found between sexes in 30-day mortality. In the same analysis, a history of preoperative diabetes and COPD had an effect on mortality. This however did not seem to affect outcome in females, who more frequently presented with a history of COPD.

Extensive aortic repair including aortic root and/or valve replacement and/or aortic arch repair may also influence outcome prognosis in patients. Nevertheless, and in contrast to the NORCAAD, intraoperative characteristics were not reported in most of the previous studies on sex differences in ATAAD. 4 9 10 11 In this study, HCA time were shorter among females. This could be due to the increased incidence of total arch procedures in males, although not statistically significant. Furthermore, DeBakey Type I dissections were more frequent among males, indicating a more widespread dissection of the aortic arch and distal aorta. Females less frequently underwent extensive repair with valve replacement compared with males, which most likely explains the shorter duration of operation, CPB, and aortic cross-clamp time in females. Alternatively, it could indicate that simplified procedures (supracoronary graft alone) were more often performed in females, and if so, possible sex bias. Such sex bias may have acted in a favorable direction: greater age and increased preoperative comorbidity thus counteracted by a swifter surgical procedure resulting in equalized early mortality.

A longer life span among females may have contributed to the increased occurrence of comorbidities such as hypertension. 4 The incidence of hypertension, which increases with age, is believed to play an important role in the pathogenesis of ATAAD. However, and in consistency with previous studies, the incidence of ATAAD for males was twice that for females, implying that females may be less prone to the development of ATAAD.

### Limitations and Strengths

This study included a subset of patients who reach designated tertiary referral centers alive. This is an important limitation, as a substantial proportion of patients with ATAAD who died prior to hospital admission may have been missed, thereby underestimating overall incidence and outcome. Importantly, data on the time duration from the onset of symptoms until the surgical treatment were often missing; therefore, these results have to be interpreted carefully. Differences may have been present in the surgical techniques used over time or among the different institutions; however, our multicenter study allowed us to include a large cohort of surgical patients with Stanford Type A dissection. Furthermore, the uniform health care systems in the Nordic countries ensured a relatively stable homogeneous populations for which follow-up was successfully achieved.

## Conclusions

In summary, this study, which is one of the largest to date, found no sex-related differences in early mortality following surgery for ATAAD. This is seen despite females being older and having more comorbidities, but also presenting with less widespread dissection than males. In line with previous studies, females represented only one-third of the cohort, implying that females may be better protected from the development of ATAAD.

## References

[1] Knipp B S Deeb G M Prager R L Williams C Y Upchurch G R Patel H J A contemporary analysis of outcomes for operative repair of type A aortic dissection in the United States Surgery 2007 142 04 524 528 17950344 10.1016/j.surg.2007.07.012

[2] Golledge J Eagle K Acute aortic dissection Lancet 2008 372 55 66 18603160 10.1016/S0140-6736(08)60994-0

[3] Lee T C Kon Z Cheema F H Contemporary management and outcomes of acute type A aortic dissection: an analysis of the STS adult cardiac surgery database J Card Surg 2018 33 01 7 18 29314257 10.1111/jocs.13511

[4] Suzuki T Asai T Kinoshita T Clinical differences between men and women undergoing surgery for acute Type A aortic dissection Cardiovasc Thorac Surg 2018;(February):1 7 10.1093/icvts/ivy005 29420730

[5] Fukui T Tabata M Morita S Takanashi S Gender differences in patients undergoing surgery for acute type A aortic dissection J Thorac Cardiovasc Surg 2015 150 03 581 5870 26190655 10.1016/j.jtcvs.2015.06.031

[6] Alam M Bandeali S J Kayani W T Comparison by meta-analysis of mortality after isolated coronary artery bypass grafting in women versus men Am J Cardiol 2013 112 03 309 317 23642381 10.1016/j.amjcard.2013.03.034

[7] Shroyer A L Coombs L P Peterson E D The Society of Thoracic Surgeons: 30-day operative mortality and morbidity risk models Ann Thorac Surg 2003 75 06 1855 1856 10.1016/s0003-4975(03)00179-6 12822628

[8] Nashef S AM Roques F Sharples L D Euroscore II Eur J Cardiothorac Surg 2012 41 04 734 745 22378855 10.1093/ejcts/ezs043

[9] Nienaber C A Fattori R Mehta R H Gender-related differences in acute aortic dissection Circulation 2004 109 24 3014 3021 15197151 10.1161/01.CIR.0000130644.78677.2C

[10] Maitusong B Sun H-P Xielifu D Sex-related differences between patients with symptomatic acute aortic dissection Medicine (Baltimore) 2016 95 11 e3100 26986151 10.1097/MD.0000000000003100 PMC4839932

[11] Conway B D Stamou S C Kouchoukos N T Lobdell K W Hagberg R C Effects of gender on outcomes and survival following repair of acute type A aortic dissection Int J Angiol 2015 24 02 93 98 26060379 10.1055/s-0034-1396341 PMC4452609

[12] Geirsson A Ahlsson A Franco-Cereceda A The Nordic Consortium for Acute type A Aortic Dissection (NORCAAD): objectives and design* Scand Cardiovasc J 2016 50(5–6):334 340 27615395 10.1080/14017431.2016.1235284

[13] Hansson E C Dellborg M Lepore V Jeppsson A Prevalence, indications and appropriateness of antiplatelet therapy in patients operated for acute aortic dissection: associations with bleeding complications and mortality Heart 2013 99 02 116 121 23048167 10.1136/heartjnl-2012-302717

[14] Harris K M Strauss C E Eagle K A Correlates of delayed recognition and treatment of acute type A aortic dissection: the International Registry of Acute Aortic Dissection (IRAD) Circulation 2011 124 18 1911 1918 21969019 10.1161/CIRCULATIONAHA.110.006320

[15] Chemtob R A Moeller-Soerensen H Holmvang L Olsen P S Ravn H B Outcome after surgery for acute aortic dissection: influence of preoperative antiplatelet therapy on prognosis J Cardiothorac Vasc Anesth 2017 31 02 569 574 28017673 10.1053/j.jvca.2016.10.007

[16] Melvinsdottir I H Lund S H Agnarsson B A Sigvaldason K Gudbjartsson T Geirsson A The incidence and mortality of acute thoracic aortic dissection: results from a whole nation study Eur J Cardiothorac Surg 2016 50 06 1111 1117 27334108 10.1093/ejcts/ezw235

[17] Howard D PJ Banerjee A Fairhead J F Perkins J Silver L E Rothwell P M Population-based study of incidence and outcome of acute aortic dissection and premorbid risk factor control: 10-year results from the Oxford Vascular Study Circulation 2013 127 20 2031 2037 23599348 10.1161/CIRCULATIONAHA.112.000483 PMC6016737

[18] Trimarchi S Eagle K A Nienaber C A Role of age in acute type A aortic dissection outcome: report from the International Registry of Acute Aortic Dissection (IRAD) J Thorac Cardiovasc Surg 2010 140 04 784 789 20176372 10.1016/j.jtcvs.2009.11.014

