# Supplementary material for: Effects of Sex on Early Outcome following Repair of Acute Type A Aortic Dissection: Results from The Nordic Consortium for Acute Type A Aortic Dissection (NORCAAD)
Source: Aorta (Stamford). 2019 Jul 22;7(1):7–14. doi: 10.1055/s-0039-1687900 (PMC6645907; doi:10.1055/s-0039-1687900)
Supplement: Supplementary file 1 — Supplementary Material [file 10-1055-s-0039-1687900-s180046.pdf]

Supplementary Table S1 Event rates with 95% CI obtained from Poisson regression for death from surgery and from discharge by sex and age group

		Men		Women	
**Endpoint**	**Age group**	***n*** **(%)**	**Event rate (95% CI) per 100 person-years**	***n*** **(%)**	**Event rate(95% CI) per 100 person-years**
Death (from surgery)	All patients	220 (28.2)	8.97 (7.86–10.24)	111 (29.8)	10.08 (8.36–12.15)
	≤ 70 y	150 (24.0)	7.32 (6.24–8.59)	64 (26.8)	8.41 (6.57–10.77)
	> 70 y	70 (44.6)	17.37 (13.74–21.96)	47 (35.1)	13.74 (10.32–18.29)
Death (from discharge)	All patients	90 (14.0)	3.75 (3.05–4.61)	49 (16.0)	4.62 (3.50–6.12)
	≤ 70 y	56 (10.7)	2.80 (2.15–3.64)	25 (12.6)	3.43 (2.32–5.08)
	> 70 y	34 (28.1)	8.56 (6.11–11.97)	24 (22.2)	7.26 (4.86–10.82)

Abbreviations: CI, confidence interval; IRR, incidence rate ratio.
Note: Event rates per 100 person-years and IRRs are obtained from Poisson regression.

Supplementary Table S2 Age-adjusted Cox proportional hazard models for prediction of time to death from discharge

Predictor	Value	Missing data	Events *n* (%)	Hazard ratio (95% CI)	*p*-Value	*p*-Value for assumption of pH
Gender	Male	0	90 (14.0)			0.38
	Female		49 (16.0)	0.94 (0.66–1.34)	0.72	
Gender (multivariable-adjusted[a])	Male	26	87 (13.9)			0.38
	Female		49 (16.5)	1.00 (0.70–1.44)	0.98	
Age (y)	Risk by 5-unit increase	0	139 (14.6)	1.35 (1.24–1.47)	< 0.0001	0.70
Age above 70 y	No	0	81 (11.2)			0.47
	Yes		58 (25.3)	2.63 (1.87–3.68)	< 0.0001	
Hypertension	No	1	68 (14.4)			0.13
	Yes		71 (14.8)	1.02 (0.73–1.43)	0.89	
Hypercholesterolemia	No	3	124 (14.7)			0.13
	Yes		15 (14.3)	0.80 (0.47–1.37)	0.42	
Diabetes	No	4	137 (14.7)			0.55
	Yes		2 (12.5)	0.75 (0.19–3.04)	0.69	
COPD	No	2	125 (13.8)			0.84
	Yes		14 (30.4)	1.72 (0.99–3.00)	0.054	
HxStroke	No	2	131 (14.4)			0.57
	Yes		8 (21.6)	1.30 (0.63–2.66)	0.47	
HxTIA	No	2	138 (14.8)			0.36
	Yes		1 (7.1)	0.57 (0.08–4.11)	0.58	
HxCKD	No	2	132 (14.2)			0.83
	Yes		7 (41.2)	2.94 (1.37–6.29)	0.0055	
Marfan syndrome	No	2	136 (15.0)			0.27
	Yes		3 (7.5)	0.60 (0.19–1.91)	0.39	
Cardiac arrest	No	2	133 (14.6)			0.29
	Yes		6 (17.1)	1.13 (0.50–2.56)	0.77	
Hypotensive shock at presentation	No	72	98 (14.1)			0.041
	Yes		29 (15.7)	0.97 (0.64–1.48)	0.90	
Pericardial tamponade	No	22	103 (13.1)			0.052
	Yes		33 (23.4)	1.51 (1.02–2.24)	0.042	
DeBakey class	I	2	109 (15.7)			0.080
	II		30 (11.8)	0.66 (0.44–1.00)	0.051	
Penn class	Penn class A	7	88 (14.1)			0.0036
	Penn class B		31 (15.7)	1.18 (0.78–1.78)	0.43	
	Penn class C		13 (15.3)	1.09 (0.61–1.95)	0.78	
	Penn class B and C		7 (18.9)	1.63 (0.76–3.53)	0.21	
CPB time (min)	Risk by 30-unit increase	66	131 (14.8)	1.02 (0.94–1.10)	0.70	0.0022

Abbreviations: CI, confidence interval; CKD, chronic kidney disease; COPD, chronic obstructive pulmonary disease; CPB, cardiopulmonary bypass; Hx, history of; TIA, transient ischemic attack.
[a]Adjusted for age, COPD, HxCKD, pericardial tamponade, and DeBakey class 2018–06–18 Analys.sas.
